# A novel *PDGFRB* sequence variant in a family with a mild form of primary familial brain calcification: a case report and a review of the literature

**DOI:** 10.1186/s12883-019-1292-8

**Published:** 2019-04-12

**Authors:** Stine Westergaard Mathorne, Kristina Sørensen, Christina Fagerberg, Matthias Bode, Jens Michael Hertz

**Affiliations:** 1 Department of Clinical Genetics, Odense University Hospital, J. B. Winsløvs Vej 4, DK-5000 Odense, Denmark; 2 Department of Neurology, Odense University Hospital, Odense, Denmark; 3 Department of Clinical Research, University of Southern Denmark, Odense, Denmark

**Keywords:** PDGFRB, Fahr syndrome, Primary familial brain calcification

## Abstract

**Background:**

Primary familial brain calcification is a rare autosomal dominant or recessive neurodegenerative disease, characterized by bilateral brain calcifications in different areas of the brain. It is a clinically heterogeneous disease and patients are reported to exhibit a wide spectrum of neurological and psychiatric symptoms. Mutations in five genes have been identified so far including *SLC20A2, PDGFRB, PDGFB, XPR1*, and *MYORG*. *PDGFRB* encodes the platelet-derived growth factor receptor-beta, and is expressed in neurons, vascular smooth muscle cells and pericytes. Patients with a *PDGFRB* mutation seem to exhibit a milder phenotype and milder brain calcification on brain imaging than patients with *SLC20A2* and *PDGFB* mutations. However, this is based on a few observations so far.

**Case presentation:**

We present a Danish family with bilateral brain calcifications and mild clinical symptoms of primary familial brain calcification, segregating with a novel *PDGFRB* sequence variant: c.1834G > A; p.(Gly612Arg), detected by whole exome sequencing. The variant results in physiochemical changes at the amino acid level, and affects a highly conserved nucleotide as well as amino acid. It is located in the tyrosine kinase domain of PDGFRβ. Segregation analysis and in silico analyses predicted the missense variant to be disease causing.

**Conclusion:**

Our study confirms that *PDGFRB* mutation carriers in general have a mild clinical phenotype, and basal ganglia calcifications can be detected by a CT scan, also in asymptomatic mutation carriers.

## Background

Primary familial brain calcification (PFBC) is a rare progressive neurodegenerative disease, previously known as Fahr’s disease or idiopathic basal ganglia calcification. It is inherited in an autosomal dominant as well as an autosomal recessive manner, and is characterized by bilateral calcifications in the basal ganglia and other brain regions including the cerebellum, thalamus and the brain stem [1]. Brain calcification is a common finding in older people [2, 3] and can be a secondary manifestation of different diseases [4]. However, the calcification in PFBC is more severe than what is expected during normal aging.

PFBC is a clinically heterogeneous disease. Patients are reported to exhibit a wide spectrum of neurological and/or psychiatric symptoms. Most frequent symptoms seem to be movement disorders, psychiatric symptoms, and cognitive impairment, although some affected individuals remain asymptomatic [1, 5].

Approximately, no more than one fourth of individuals with a clinical diagnosis of PFBC are found to have a heterozygous pathogenic variant in one of the four genes known to cause autosomal dominant PFBC: *SLC20A2, PDGFRB, PDGFB* and *XPR1*, and some may have mutations in the *MYORG* gene related to autosomal recessive PFBC [6].

*PDGFRB* is located at 5q32 and encodes the receptor for platelet-derived growth factor beta (PDGF-B). PDGFRβ (Platelet-Derived Growth Factor Receptor Beta) is expressed in neurons, vascular smooth muscle cells and pericytes [7–9]. There seems to be a tendency of a mild phenotype and a high proportion of asymptomatic individuals in patients with *PDGFRB* variants compared to patients with *SLC20A2* and *PDGFB* variants [1, 5].

Ten different sequence variants in *PDGFRB* in patients with PFBC have been reported so far [10]. A number of other phenotypes have been associated with sequence variants in *PDGFRB* including infantile myofibromatosis and Kosaki overgrowth syndrome [11–13].

We detected a novel *PDGFRB* missense variant by exome sequencing in a Danish family with bilateral brain calcifications and mild clinical symptoms of PFBC. Segregation analysis of the family could demonstrate that basal ganglia calcifications can be detected by a CT scan, also in asymptomatic mutation carriers.

## Case presentation

The proband (III:1) is a 58-year-old woman with paresthesia and tendency to muscle cramps in the lower legs and feet.

She was referred to a neurologist at the age of 56 because of a sensation of warmth/cold in the lower legs and tendency to cramp in the lower legs, especially the feet. Neurological examination was normal. She was born with atrial septal defect (ASD), has had rheumatoid arthritis since the age of 28 years, has hypercholesterolemia, and recently she was diagnosed with anxiety.

MRI of the brain showed bilateral calcifications in the globus pallidus, putamen, thalamus and nucleus dentatus, as well as diffuse white matter lesions in both cerebral hemispheres consistent with chronic ischemia (leukoaraiosis). Cerebral computerized tomography (CT) scan also showed bilateral calcifications in the basal ganglia and cerebellum, and distinct periventricular leukoaraiosis.

The probands mother (II:2), have paroxysmal atrial fibrillation, and was admitted to hospital at the age of 74 years suspected of having transient ischemic attack. She presented with sudden blindness on both eyes and dizziness, lasting for about 3–4 min. Neurological examination showed decreased vibratory sensation, and the Achilles reflexes was absent bilateral. MRI of the brain showed distinct leukoaraiosis due to ischemic demyelination. CT scan was not performed.

The monozygotic twin sister of the proband (III:2) was admitted to hospital at the age of 49 years because of paresthesia on the left side. Since her mid-fifties she has had sore muscles in all four extremities. At the time of diagnosis, she suffered from restless legs syndrome with an urge to move the legs, unpleasant sensations in her legs and sometimes in the hands as well, totally relieved by the movement. Neurological examination was normal except slight insecurity at Rombergs test. A CT scan showed bilateral calcifications in the basal ganglia and cerebellum, extensive periventricular leukoaraiosis. Magnetic resonance imaging (MRI) showed white matter lesions in both cerebral hemispheres and the brain stem. She was also born with ASD. The two monozygotic twin sisters are concordant with respect to the age of onset.

A younger sister (III:3), who suffers from epilepsy, was admitted to hospital at the age of 48 years because of transient dizziness and hemiparesis at the right side. Both CT scan and brain MRI showed bilateral calcifications in the basal ganglia and cerebellar areas, as well as distinct leukoaraiosis.

**Fig. 1:**
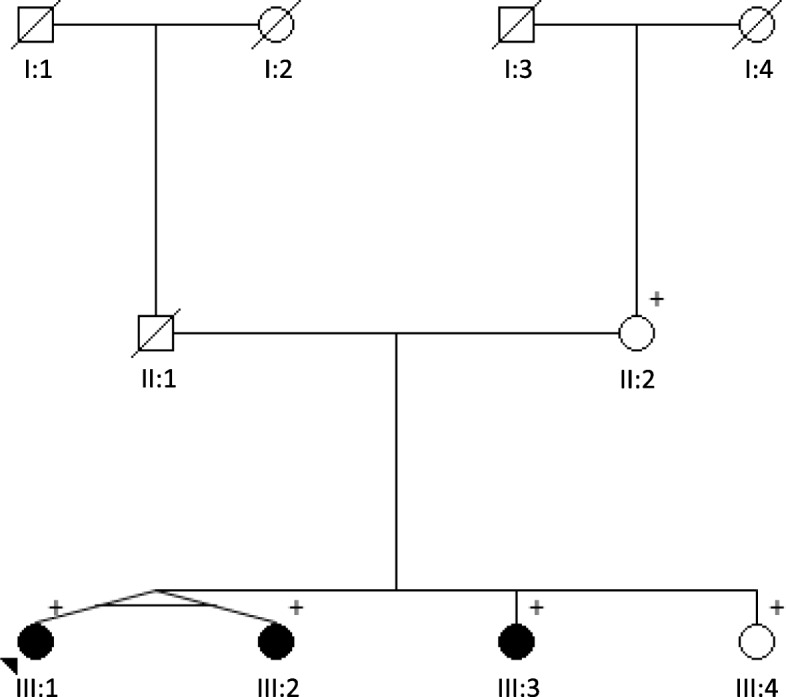
Pedigree of the family. The proband is marked with an arrow, filled symbols indicate individuals with brain calcification on CT, + indicate mutation carriers

The probands youngest sister (III:4) is clinically asymptomatic. Nevertheless, she has bilateral calcifications in the basal ganglia and in the nucleus dentatus on her CT scan (Table 1, Fig. 1).

**Table 1:** Clinical features and imaging findings of family members heterozygous for the *PDGFRB* sequence variant

Patient	Age at onset of clinical symptoms, years	Age at evaluation, years	Clinical features	CT scan	MRI
II:2	NA	74	TIA Atrial fibrillation Decreased vibratory sensation	ND	Leukoaraiosis
III:1	54	56	Anxiety Paresthesia Tendency to cramp in feet. Congenital ASD	Basal ganglia, cerebellum, distinct periventricular leukoaraiosis.	Pa, Pu, T, D Diffuse white matter lesions at temporal horns, corona radiata, centrum semiovale, subcortical and periventricular consistent with chronic ischemia
III:2	49	56	Paresthesia Sore muscles Congenital ASD	Basal ganglia, cerebellum, distinct leukoaraiosis	WM lesions in both cerebral hemispheres and brain stem
III:3	48	48	Epilepsy Transient dizziness and hemiparesis	Basal ganglia and cerebellar areas	ND
III:4		52	None	Ca, lentiformis, D	ND

Abbreviations: *NA* = not applicable, *ND* = not done, *Ca* = caudate calcifications, *D* = dentate calcifications, *Pa* = globus pallidus calcifications, *Pu* = putamen calcifications, *T* = thalamic calcifications; *TIA* = transient ischemic attack

All calcifications mentioned in the table are bilateral

DNA from the proband and her mother was extracted from EDTA-stabilized peripheral blood lymphocytes and subjected to exome capture using NimbleGen SeqCap EZ MedExome (Roche), followed by sequencing on an Illumina NextSeq550 platform to a mean coverage of 140x, with 95% of targeted bases covered with minimum 30x coverage. Raw reads were aligned using the Burrows-Wheeler Alignment tool v. 0.7.15 [14] and the GATK (Genome Analysis Toolkit) Best Practice pipeline v. 3.8–0 was used for variant calling [15]. Annotation and filtering of variants was performed using VarSeq 2.0.2 (Golden Helix). The sequence variant has been confirmed by bidirectional Sanger sequencing of *PDGFRB* (NM_002609.3) according to standard procedures.

Whole exome sequencing on DNA from the proband and her mother, and bidirectional Sanger sequencing of *PDGFRB* in III:2, III:3 and III:4*,* showed heterozygosity for a novel sequence variant, c.1834G>A; p.(Gly612Arg) in *PDGFRB.* The sequence variant is located in exon 13 within the tyrosine kinase domain [Table 2], and can be classified as likely pathogenic class 4 variant according to the American College of Medical Genetics and Genomics and the Association for Molecular Pathology [16]. A CADD score of 29.5 further support the pathogenicity of the variant [17].

**Table 2:** Variants reported in *PDGFRB* and associated with PFBC

Nucleotide substitution (Ref.)	Amino acid substitution	Exon	Domain affected	Global allele frequency (gnomAD)	In silico analysis
c.3G>A [30]	p.(Met1Ile)	2	Initiation codon	No data	SIFT: damaging MutationTaster: polymorphism PolyPhen-2: benign
c.676C>T [31]	p.(Arg226Cys)	5	Extracellular, Ig-like C2-type 3	No data	SIFT: deleterious MutationTaster: disease causing PolyPhen-2: probably damaging
c.1126C>T [32]	p.(Arg376Trp)	7	Extracellular, Ig-like C2-type 4	0.00000829/2	SIFT: deleterious MutationTaster: disease causing PolyPhen2: probably damaging
c.1787C>T [31]	p.(Pro596Leu)	12	Tyrosine kinase domain	0.00080/2	SIFT: deleterious MutationTaster: disease causing PolyPhen-2: probably damaging
c.1834G>A (*)	p.(Gly612Arg)	13	Tyrosine kinase domain	No Data	SIFT: deleterious MutationTaster: disease causing PolyPhen-2: probably damaging
c.1973 T>C [27]	p.(Leu658Pro)	14	Tyrosine kinase domain	No data	SIFT: deleterious MutationTaster: disease causing PolyPhen-2: probably damaging
c.2083C>T [22]	p.(Arg695Cys)	15	Tyrosine kinase domain	0.000110/27	SIFT: deleterious MutationTaster: disease causing PolyPhen-2: probably damaging
c.2209G>A [30]	p.(Asp737Asn)	16	Tyrosine kinase domain	0.00000406/1	SIFT: Tolerated MutationTaster: disease causing PolyPhen-2: probably damaging
c.2531A>G [31]	p.(Asp844Gly)	18	Tyrosine kinase domain	No data	SIFT: deleterious MutationTaster: disease causing PolyPhen-2: probably damaging
c.2959C>T [27]	p.(Arg987Trp)	22	–	0.0000285/7	SIFT: deleterious MutationTaster: disease causing PolyPhen-2: probably damaging
c.3212A>T [5]	p.(Glu1071Val)	23	–	No data	SIFT: Tolerated MutationTaster: disease causing PolyPhen-2: possibly damaging

^*^Detected in the present study

The clinical findings in the present family are compatible with the phenotype associated with variants in *PDGFRB* (Fig. 1, Table 1).

## Discussion and conclusion

**Fig. 2:**
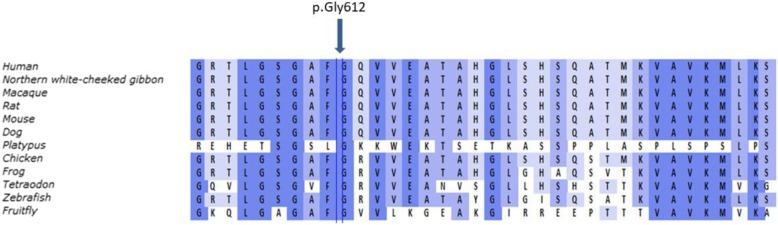
Evolutionary alignments of the affected amino acid in PDGFRB. Amino acids conserved relative to the human sequence are shaded in blue

We present a family with mild clinical signs of PFBC, in which a not previously reported heterozygous sequence variant in *PDGFRB* was found to segregate. The variant, c.1834G>A; p.(Gly612Arg), is located in exon 13 and results in a glycine being substituted by an arginine in the tyrosine kinase domain of PDGFRβ. Glycine at amino acid position 612 in PDGFRβ is evolutionary conserved to *Fruit fly* (Fig. 2). Conversion to arginine results in a physiochemical change, and is predicted to be disease causing by the in silico program MutationTaster [18], deleterious by SIFT [19] and probably damaging by PolyPhen-2 [20]. It is most likely that heterozygosity for this variant explains the phenotype of the affected family members, who presented with a mild clinical phenotype of PFBC, but widespread calcifications on a CT-scan.

PDGFRβ is a cell-surface tyrosine kinase receptor. It consists of an intracellular tyrosine-kinase domain and five extracellular immunoglobulin loops [21]. Activation of PDGFRβ results in dimerization of the receptor and autophosphorylation of tyrosine residues. This further activates downstream signaling pathways which mediates cellular proliferation, differentiation, survival, and migration [21].

The intracellular protein tyrosine kinase domain mediates the intrinsic functions of the activated PDGFRβ receptor, thus altered phosphorylation at the tyrosine kinase domain may induce impaired signaling in the PDGF-B/PDGFRβ pathway. A functional study concluded that missense variants in the tyrosine kinase domain of PDGFRβ directly interfere with autophosphorylation of the PDGFRβ receptor [22]. Furthermore, several studies have shown that in patients with PFBC, *PDGFB* and *PDGFRB* variants lead to decreased PDGF-B/PDGFRβ signaling [22–24]. In contrast, increased signaling is associated with cancers, infantile myofibromatosis, Kosaki overgrowth syndrome and Penttinen syndrome [11–13, 25, 26].

The amino acid substitution, p.(Gly612Arg), as detected in all family members with CT scans in accordance with a diagnosis of PFBC in the present family, is located in the intracellular tyrosine kinase domain, and is predicted to impair the PDGF-B/PDGFRβ pathway.

Two pathophysiological hypotheses for the molecular mechanism of PFBC caused by *PDGFRB* mutations have been suggested. Loss of function of *PDGFRB* could induce calcium depositions in the brain, by impairing the integrity of the blood-brain barrier. This hypothesis might be supported by the functional studies, who found that *PDGFRB* variants and *PDGFB* variants associated with PFBC results in loss/reduced function of the gene product [22–24]. Another hypothesis suggests that an activating mutation could induce brain calcifications directly through influence of the PDGF-Pit-1 pathway [27]. Although different hypotheses have been suggested, it is not yet clear how impaired PDGF-B/PDGFRβ signaling leads to microvascular calcification in the brain [24, 27].

In patients with PFBC, expressivity is highly variable in symptoms, age of onset (median 31 years, range 6–77 years) [28] and severity of symptoms, even within the same family. A systematic review by Tadic et al., showed that the penetrance of the imaging phenotype is 100%, however the penetrance of the clinical phenotype is reduced to 61% [1]. Especially patients with a *PDGFRB* mutation seem to exhibit a mild clinical phenotype and have the highest proportion of asymptomatic individuals [1]. This is consistent with the findings in the present study, where family members with the sequence variant presented with a mild clinical phenotype of PFBC with few or no neurological symptoms, but all who were evaluated with a CT scan presented with calcifications on brain imaging (Table 1).

According to Nicolas et al., no correlation is found between location of calcification and symptoms, nor between the extent of calcification and the severity of symptoms [5]. However, it has been observed that the severity of calcifications is higher in symptomatic versus asymptomatic people [5, 29]. Furthermore, it has been reported that *PDGFRB* mutation carriers seem to have a milder brain calcification [28].

In conclusion, we report a novel heterozygous missense variant, c.1834G>A; p.(Gly612Arg) in *PDGFRB* in a family with a mild form of PFBC. Our study confirms that *PDGFRB* mutation carriers in general may have a mild clinical phenotype, and basal ganglia calcifications can be detected by a CT scan also in asymptomatic mutation carriers.

## Abbreviations

CADD: Combined Annotation-Dependent Depletion
CT: Computerized tomography
DNA: Deoxyribonucleic acid
EDTA: Ethylenediaminetetraacetic acid
GATK: Genome Analysis Toolkit (variant calling software)
HGMD: Human Genome Mutation Database
MRI: Magnetic resonance imaging
PDGF-B: Platelet-derived growth factor beta (gene/protein name)
PDGFRβ: Platelet-derived growth factor receptor beta (gene/protein name)
PFBC: Primary familial brain calcification
SIFT: Sorting Intolerant From Tolerant (name of a prediction software)
SLC20A2: Solute Carrier Family 20, member 2 (gene/protein name)
XPR1: Xenotropic and Polytropic Retrovirus Receptor (gene/protein name)
